# DHEA Supplementation Confers No Additional Benefit to that of Growth Hormone on Pregnancy and Live Birth Rates in IVF Patients Categorized as Poor Prognosis

**DOI:** 10.3389/fendo.2018.00014

**Published:** 2018-01-31

**Authors:** Kevin N. Keane, Peter M. Hinchliffe, Philip K. Rowlands, Gayatri Borude, Shanti Srinivasan, Satvinder S. Dhaliwal, John L. Yovich

**Affiliations:** ^1^School of Pharmacy and Biomedical Science, Faculty of Health Sciences, Curtin University, Perth, WA, Australia; ^2^PIVET Medical Centre, Perth, WA, Australia; ^3^Faculty of Health Sciences, School of Public Health, Curtin University, Perth, WA, Australia

**Keywords:** growth hormone, dehydroepiandrosterone, *in vitro* fertilization, embryo quality, adjuvants

## Abstract

**Background:**

*In vitro* fertilization (IVF) patients receive various adjuvant therapies to enhance success rates, but the true benefit is actively debated. Growth hormone (GH) and dehydroepiandrosterone (DHEA) supplementation were assessed in women undergoing fresh IVF transfer cycles and categorized as poor prognosis from five criteria.

**Methods:**

Data were retrospectively analyzed from 626 women undergoing 626 IVF cycles, where they received no adjuvant, GH alone, or GH–DHEA in combination. A small group received DHEA alone. The utilization of adjuvants was decided between the attending clinician and the patient depending on various factors including cost.

**Results:**

Despite patients being significantly older with lower ovarian reserve, live birth rates were significantly greater with GH alone (18.6%) and with GH-DHEA (13.0%) in comparison to those with no adjuvant (*p* < 0.003). No significant difference was observed between the GH groups (*p* = 0.181). Overall, patient age, quality of the transferred embryo, and GH treatment were the only significant independent predictors of live birth chance. Following adjustment for patient age, antral follicle count, and quality of transferred embryo, GH alone and GH–DHEA led to a 7.1-fold and 5.6-fold increase in live birth chance, respectively (*p* < 0.000).

**Conclusion:**

These data indicated that GH adjuvant may support more live births, particularly in younger women, and importantly, the positive effects of GH treatment were still observed even if DHEA was also used in combination. However, supplementation with DHEA did not indicate any potentiating benefit or modify the effects of GH treatment. Due to the retrospective design, and the risk of a selection bias, caution is advised in the interpretation of the data.

## Introduction

Assisted reproductive technology (ART) incorporating *in vitro* fertilization (IVF) are key strategies to increase the possibility of conceiving for individuals who have experienced difficulties with natural conception. As a result of socioeconomic factors in both Western and Asian cultures, there is an increasing propensity for males and females to delay childbirth until an older age. Due to the natural process of decreased fertility with age, this has meant that increasing numbers of patients are seeking support from fertility specialists. In addition, with the global increase in endocrine disorders including obesity and metabolic syndrome, along with other ovarian pathologies that affect fertility, for instance endometriosis, adenomyosis and leiomyomata, patient management is challenged over and above the impact of increasing age. Historically, a subgroup of patients defined as poor prognosis according to the Bologna criteria (1) have proved very difficult to treat and have poor outcomes. They possess a combination of characteristics such as advanced maternal age (40 years and above), poor response to standard gonadotropin stimulation (less than four oocytes on collection), and/or a minimal ovarian reserve, as evident by a low anti-Mullerian hormone (AMH) level (less than 0.5–1.1 ng/ml; 3.7–8.1 nmol/l) or low antral follicle count (AFC; less than 5–7 follicles). However, these criteria do not include patients who produce poor quality embryos for unknown reasons and are equally considered poor prognosis. Nonetheless, ART clinics are faced with an increased demand for improved treatment, and one mechanism to address this, but which has proved highly controversial, has been to include off-label adjuvant therapy as a part of IVF management.

Two of the most common IVF adjuvant therapies are growth hormone (GH) and dehydroepiandrosterone (DHEA) supplementation (2). However, as a consequence of various investigations that have been either poorly designed or utilized small patient cohorts, along with a lack of sufficiently powered randomized controlled trials (RCTs) in this area, the true beneficial effects of GH and DHEA are actively debated and still remain clinically unclear (2). GH is an anabolic peptide hormone that functions to increase cell proliferation and growth, and it has been demonstrated to improve the sensitivity to gonadotropin stimulation and boost oocyte yield in several IVF studies (3–9). Recently, we (10) along with others (9, 11–15) found that GH promoted pregnancy rate and/or live birth chance by reducing miscarriage rates (13, 16, 17). It is hypothesized that GH reduces aneuploidies by enhancing embryo quality, and some evidence indicated that it may have a role in this respect (14), but the molecular mechanism is not understood. On the other hand, numerous other studies (5, 18–23), along with some meta-analyses and systematic reviews (7, 8), have demonstrated that GH has no clear positive effect on pregnancy or live birth outcomes in IVF patients. Similarly, DHEA, a multifunctional adrenal prohormone that acts as a precursor for testosterone and estradiol synthesis (24), has also been reported to promote enhanced pregnancy and live birth rates in poor prognosis IVF patients (25–34). In addition, DHEA has been suggested to increase fertilization rates and embryo quality (27, 32, 35, 36). Equally, other investigations including meta-analyses (15, 37–39) have demonstrated no clear benefit in terms of oocytes collected, embryo quality, or clinical pregnancy and live birth rates (30, 40–43). Thus, the use of these adjuvant therapies in IVF remains highly controversial and inconclusive (2).

These hormones are believed to modulate ovarian physiology, including oocyte and follicle maturation, and could have local effects on the endometrium during ovulation and implantation (37, 38, 44). To date, very little research, RCT or observational, has investigated the synergistic effects of GH and DHEA in combination in the IVF setting (45). Following on from our previous 2017 retrospective investigation where we found that GH in isolation enhanced clinical outcomes (10), we aimed to determine whether the addition of DHEA to treatment cycles resulted in any further beneficial outcomes. Consequently, in this study, we examined the effect of (+)GH alone, (+)DHEA alone, and (+)GH–DHEA in combination on poor prognosis IVF patients and primary outcomes included pregnancy and live birth rates. As a secondary aim, due to the controversy surrounding the use of IVF adjuvants and the definition of poor prognosis patients, we intended to identify significant variables that should be considered when designing future prospective studies in this research area. Therefore, we included a broader definition of “poor prognosis” IVF patients, which extended the Bologna criteria to those with repetitive implantation failure (RIF) and those who generated lower quality embryos.

## Materials and Methods

### Study Period and Participants

This retrospective study covered a period from 1 April 2008 to 31 December 2015 and is subsequent to another study investigating GH administration in isolation (10). The current analysis specifically focused on a subset of patients who were offered IVF adjuvants at any point in their treatment history by attending clinicians, because they were classified as poor prognosis with one or more of the following criteria: (i) women with fewer than four metaphase II (M II) oocytes although receiving maximal FSH stimulation (i.e., 450 IU/day); (ii) women where the majority of embryos were graded poor quality with marked fragmentation (>50%) (46); (iii) women with repetitive fresh or frozen embryo transfers (≥3 transfers) without pregnancy; (iv) women aged ≥40 years who had at least one failed IVF cycle; and (v) women with ≤8 antral follicles. The data collection, storage, and analysis were conducted independently from the clinicians who conducted the consultations, IVF procedures, and prescribed adjuvants.

**Figure 1:**
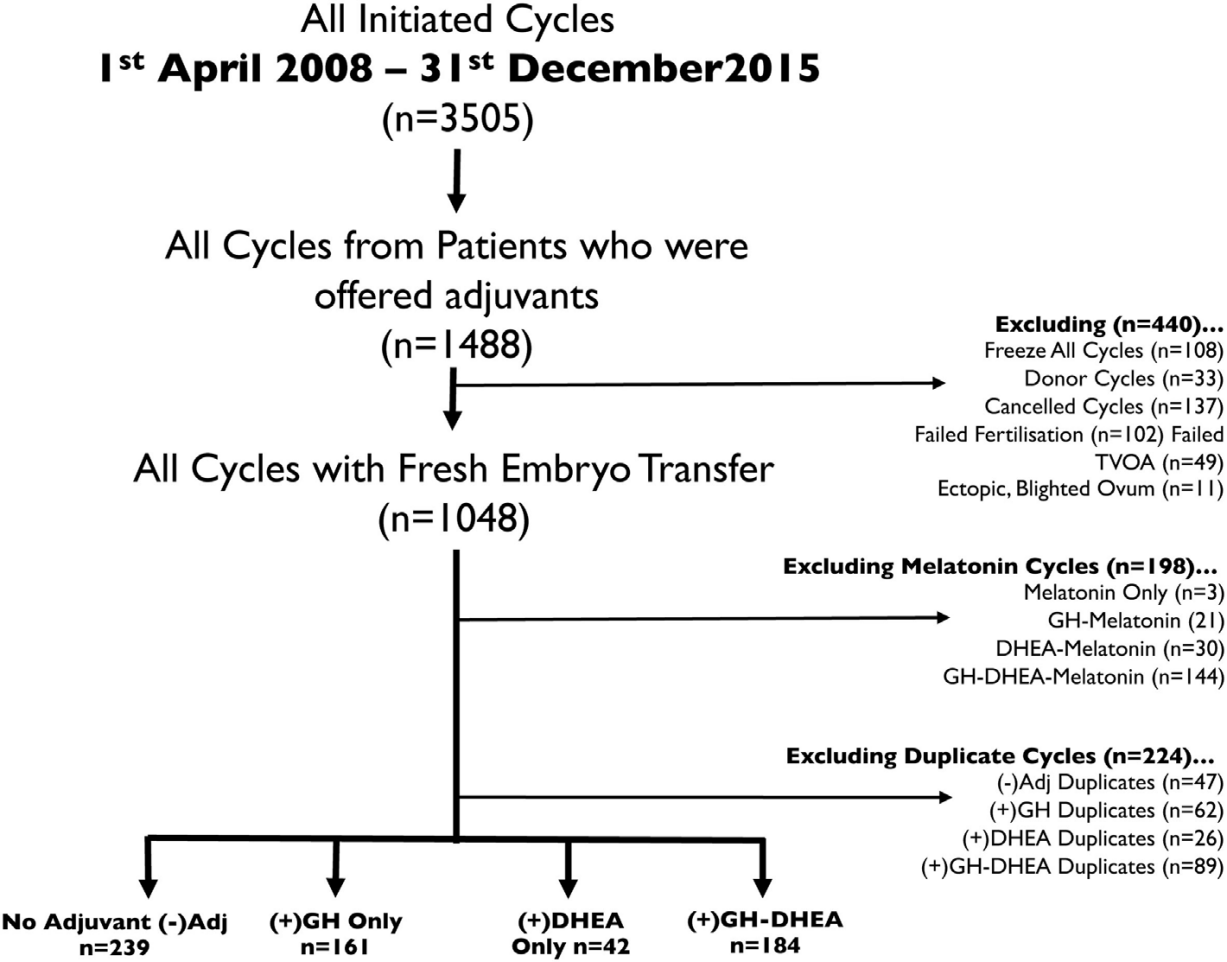
Flow diagram of data extraction. Data were extracted from the PIVET database, and cases/cycles removed on the basis of cycle outcome (e.g., canceled/donor), melatonin treatment, and cycle type (failed transvaginal oocyte aspiration, failed fertilization, or freeze all).

To limit the bias of including multiple treatment cycles for individual patients and in an attempt to randomize cases, only the first IVF cycle with successful ovum pick-up (OPU) and fresh embryo transfer (ET) for each patient during the study period was included in the analysis. Furthermore, only cycles where either no adjuvant therapy, GH-only, DHEA-only, or GH and DHEA in combination was administered were included. This meant that cycles with other adjuvant such as melatonin were excluded. In total, 626 eligible women had 626 IVF cycles that resulted in successful OPU and fresh ET. A total of 239 cycles/women had their first cycle within the time period that was free from any adjuvant intervention, designated (−)Adj. A total of 161 different women had 161 cycles with GH alone (+)GH, while 42 others received DHEA alone (+)DHEA, and 184 received GH and DHEA in combination (+)GH–DHEA (Figure 1). Each cycle/woman included in the analysis represented their first initiated IVF cycle within the study period but not necessarily their first in their treatment history. Patients elected to use GH, DHEA, or the combination based on the several factors, one of which was expense (since patients were charged for the drugs).

### Clinical Management

Growth hormone (Saizen) was administered during the preceding menstrual cycle commencing on day 2–3, thereafter the injection of six vials of 9 IU over 6 weeks in the lead-up to OPU, and equated to 54 IU over 33–37 days averaging approximately 1.5 IU per day. SciTropin (0.3 mg) was injected daily for 45 days prior to trigger, with patients receiving GH at precisely 1.0 IU per day up to OPU (10). For DHEA administration, each patient utilized one half of a DHEA troche containing 25 mg of micronised DHEA sublingual twice daily, commencing in the preceding menstrual cycle and given over a period of 6 weeks in the lead-up to OPU (47). Where GH was administered in combination with DHEA, the two adjuvants were given over the same 6-week period.

All patients were stimulated with recombinant FSH using specific dosage algorithms as defined recently (48) and in most cases (43.9% of cycles) using an antagonist protocol (Orgalutran). Older women with a low AFC rating received a flare-agonist regimen (37.1%) or specialized downregulation protocols (19.0%) (49) (Table 1). These stimulation protocols applying the PIVET dosing algorithm have been well described with minimized OHSS risk and fewer than 4% of cases overall generating more than 20 oocytes (48). Ovulation was triggered with human chorionic gonadotrophin (HCG). OPU was performed using transvaginal oocyte aspiration and undertaken 36 h posttrigger with IV sedation and using a double-lumen flushing/aspiration needle (Cook, Australia). The luteal phase was managed using HCG support (50). Additional support hormones were given as required (estradiol, progesterone, or combined estradiol/progesterone pessary). Where ≥12 oocytes were recovered, progesterone pessaries replaced HCG injections.

**Table 1:** Overview of main parameters that affect clinical pregnancy and live birth rates.

	No clinical pregnancy	Yes clinical pregnancy		Totals	*p* value
		No live birth	Yes live birth		
Number of cycles	534	27	65	626	
Age, years (SD)	39.0 ± 4.2	38.3 ± 3.7	36.6 ± 4.1^a^	–	<0.000
AMH, pmol/l (SD)	8.0 ± 9.8	9.0 ± 11.3	10.5 ± 12.7	–	0.521
BMI, kg/m^2^ (SD)	24.2 ± 4.8	26.8 ± 4.9^a^	24.5 ± 4.2	–	0.017
Embryos transferred, N (SD)	1.46 ± 0.51	1.52 ± 0.51	1.52 ± 0.50	–	0.589
Oocytes retrieved, N (SD)	6.8 ± 4.4	7.6 ± 5.0	8.0 ± 4.6	–	0.078
Oocyte utilization rate, % (SD)	40.6 ± 25.5	39.7 ± 26.9	36.5 ± 21.1	–	0.458
Two pronuclei generated, N (SD)	3.7 ± 2.7	4.6 ± 3.0	4.8 ± 3.4^a^	–	0.002
Fertilization rate, % (SD)	59.0 ± 23.7	62.3 ± 23.9	60.8 ± 19.1	–	0.683
Embryos cryopreserved, N (SD)	0.59 ± 1.15	0.81 ± 1.24	0.86 ± 1.33	–	0.154
Embryo utilization rate, % (SD)	69.4 ± 30.7	63.2 ± 29.6	61.7 ± 29.5	–	0.107
Percentage of high-quality embryos, % (SD)	33.3 ± 26.6	39.0 ± 29.3	43.2 ± 23.0^a^	–	0.012
Percentage of medium-quality embryos, % (SD)	39.5 ± 24.4	34.8 ± 23.6	38.2 ± 20.5	–	0.579
Percentage of low-quality embryos, % (SD)	27.2 ± 25.8	26.2 ± 28.7	18.6 ± 16.5^a^	–	0.034

**Age groups**					
<35 years	85 (15.9%)	3 (11.1%)	20 (30.8%)	108	0.001
35–39 years	170 (31.8%)	14 (51.9%)	28 (43.1%)	212	
40–44 years	253 (47.4%)	10 (37.0%)	17 (26.2%)	280	
>44 years	26 (4.9%)	0 (0.0%)	0 (0.0%)	26	

**AFC groups**					
A (>20 follicles)	56 (10.5%)	5 (18.5%)	9 (13.8%)	70	0.035
B (13–19 follicles)	78 (14.6%)	2 (7.4%)	11 (16.9%)	91	
C (9–12 follicles)	95 (17.8%)	8 (29.6%)	14 (21.5%)	117	
D (5–8 follicles)	192 (36.0%)	10 (37.0%)	25 (38.5%)	227	
E (<5 follicles)	113 (21.2%)	2 (7.4%)	6 (9.2%)	121	

**Stimulation groups**					
Antagonist	234 (43.8%)	10 (37.0%)	31 (47.7%)	275	0.532
Flare agonist	198 (37.1%)	9 (33.3%)	25 (38.5%)	232	
Other (downregulation)	102 (19.1%)	8 (29.6%)	9 (13.8%)	119	

**Adjuvant groups**					
(−)Adj	215 (40.3%)	13 (48.1%)	11 (16.9%)	239	0.118
(+)GH	126 (23.6%)	5 (18.5%)	30 (46.2%)	161	
(+)DHEA	39 (7.3%)	3 (11.1%)	0 (0.0%)	42	
(+)GH–DHEA	154 (28.8%)	6 (22.2%)	24 (36.9%)	184	

*^a^Statistically different from no clinical pregnancy group*.

*Patient age was the most significant predictor of successful clinical pregnancy or live birth rates. Also patients with a larger proportion of high-quality embryos had greater live birth rates. Key parameters such as AMH level, AFC, and stimulation protocol did not alter these rates*.

*AFC, antral follicle count; AMH, anti-Mullerian hormone; BMI, body mass index; DHEA, dehydroepiandrosterone; GH, growth hormone*.

### Embryo Culture and Assessment

Retrieved oocytes were cultured for 4–5 h postcollection prior to insemination with spermatozoa (100,000/ml) for IVF, or denuded with hyaluronidase and mature oocytes were injected using ICSI. Day 3 embryos were graded using a four-point system, with half points increments as previously published (51). Day 5 embryos were graded using the Gardner scoring system for blastocysts (52). While the clinic has a strong policy for single embryo transfer, patients categorized as poor prognosis can receive up to two day 3 embryos and such ensued in 85 (−)Adj, 93 (+)GH, 18 (+)DHEA, and 93 (+)GH–DHEA cycles, respectively. On a very rare occasion, three day 3 embryos were transferred and occurred in one cycle each for (−)Adj, (+)GH, and (+)GH–DHEA treatment. Blastocysts were transferred in a minority of these poor prognosis cycles (9.9%) as the clinic policy requires >3 high-quality embryos progressing on cleavage stage day 3 to allow culture through to day 5 or day 6.

### Data Analysis and Statistics

Primary clinical outcomes included likelihood of clinical pregnancy (presence of an intrauterine gestational sac with fetal heart beat at 7 weeks gestation) and/or live birth. Logistic regression was used to assess the independent contributions of individual confounding parameters on these outcomes such as age, body mass index (BMI), AMH level, AFC, stimulation protocol type, quality, developmental stage, and number of embryos transferred, in addition to the number of patient infertility factors and the number of previous IVF attempts. The unadjusted effect of adjuvant administration on these binary outcomes was also assessed. The effect of each variable was expressed as an odds ratio (OR) with associated 95% confidence interval (CI). Stepwise multiple logistic regression analyses enabled the determination of the minimum number of independent variables that could be used for predicting clinical pregnancy and/or live birth chance. The coefficients of the independent variables from each of the final logistic regression models were used to calculate OR and CI for clinical pregnancy and/or live birth chance due to adjuvant treatment. Continuous variables were compared using two-sample *t*-tests, and categorical variables were compared using Fisher’s Exact Chi-square tests.

## Results

### Overview of Patient Demographics and Adjuvant Treatment Groups

The majority of the cycles analyzed in this poor prognosis cohort resulted in no clinical pregnancy (85.3%), as expected for a poor prognosis cohort. The overall pregnancy rate was 14.7%, whereas the live birth rate was 10.4% (miscarriage rate of 29.3%, 27/92; Table 1). The majority of women were aged between 35 and 44 years (78.6%), with an AFC of ≤8 follicles (348/626; 55.6%), and most received antagonist stimulation (43.9%). For those who became pregnant, they tended to have more embryos cryopreserved (mean of 0.8 versus 0.6 embryos) and were significantly younger (mean age of 37.1 versus 39.0 years), generating significantly more high-quality embryos (42.0% versus 33.3%) and zygotes with two pronuclei (4.8 versus 3.7 zygotes). These trends were more pronounced for those who went on to have a successful live birth. There was no significant difference in the mean number of embryos transferred, mean oocytes retrieved, or oocyte/embryo utilization rates among those who failed to become pregnant, those who did become pregnant, those who miscarried, and those who had a live birth (Table 1).

No adjuvant (−)Adj, was administered in 38.2% of analyzed cycles, while (+)GH, (+)DHEA and (+)GH–DHEA was used in 25.7, 6.7, and 29.4% of cycles, respectively (Table 2). However, in all of the cycles where there was a live birth, 83.1% were derived from a (+)GH cycle [i.e., (+)GH and (+)GH–DHEA]. Only 16.9% of live births came from (−)Adj cycles. The largest proportion of live birth (46.2%) were from (+)GH alone cycles. Addition of DHEA did not alter this rate significantly with 36.9% of live births from cycles with GH and DHEA in combination. However, no live births were recorded in the small (+)DHEA-only group of 42 cases (Table 1). The majority of miscarriages occurred in (−)Adj cycles (48.1%; Table 1). Overall, the clinical pregnancy rate in (−)Adj, (+)GH, (+)DHEA, and (+)GH-DHEA groups was 10.0, 21.7, 7.1, and 16.3%, respectively, and the live birth rate was 4.6, 18.6, 0.0, and 13.0%, respectively (Table 2).

**Table 2:** Overview of main parameters for adjuvant treatment groups.

	(−)Adj	(+)GH	(+)DHEA	(+)GH–DHEA	*p* value
Number of cycles	239	161	42	184	–
Age, years (SD)	37.5 ± 4.3	39.1 ± 4.1^a^	39.2 ± 4.1	39.9 ± 3.9^a^	0.000
AMH, pmol/l (SD)	12.2 ± 13.3	5.3 ± 6.3^a^	4.9 ± 4.8^a^	7.0 ± 7.7^a^	0.000
BMI, kg/m^2^ (SD)	24.4 ± 4.8	24.2 ± 5.5	24.4 ± 4.5	24.3 ± 4.4	0.997
Embryos transferred, N (SD)	1.36 ± 0.49	1.59 ± 0.51^a^	1.43 ± 0.50	1.52 ± 0.51^a^	0.000
Oocytes retrieved, N (SD)	7.7 ± 4.3	7.2 ± 5.1	5.4 ± 3.3^a^	6.2 ± 4.4^a^	0.000
Oocyte utilization rate, % (SD)	33.4 ± 21.2	44.4 ± 27.0^a^	42.1 ± 23.3	44.5 ± 26.9^a^	0.000
Two pronuclei generated, N (SD)	4.2 ± 2.9	4.1 ± 5.2	3.2 ± 2.1	3.5 ± 2.9^a^	0.036
Fertilization rate, % (SD)	56.6 ± 22.9	58.9 ± 23.1	64.2 ± 23.9	62.2 ± 23.5	0.067
Embryos cryopreserved, N (SD)	0.72 ± 1.22	0.73 ± 1.29	0.38 ± 0.73	0.49 ± 1.07	0.076
Embryo utilization rate, % (SD)	61.4 ± 30.2	74.5 ± 33.0^a^	68.8 ± 28.3	71.4 ± 28.4^a^	0.000
Percentage of high-quality embryos, % (SD)	32.9 ± 24.5	37.0 ± 27.4	37.7 ± 24.6	34.0 ± 28.6	0.403
Percentage of medium-quality embryos, % (SD)	41.3 ± 22.5	38.5 ± 24.7	36.8 ± 26.0	37.1 ± 24.8	0.303
Percentage of low-quality embryos, % (SD)	25.8 ± 23.2	24.2 ± 25.1	25.5 ± 25.6	28.9 ± 27.6	0.360
Fresh embryo transfer cycles, N	239	161	42	184	–
Fresh ET pregnancy rate, N (%)	24/239 (10.0)	35/161 (21.7)	3/42 (7.1)	30/184 (16.3)	0.005
Fresh ET live birth rate, N (%)	11/239 (4.6)	30/161 (18.6)	0/42 (0.0)	24/184 (13.0)	0.000
Fresh ET miscarriage rate, N (%)	13/24 (54.2)	5/35 (14.3)	3/3 (100.0)	6/30 (20.0)	0.000

*^a^Statistically different from (−)Adj group*.

*From the complete data set, there was no significant difference between (+)GH cycles and (−)GH with regard to patient BMI; mean fertilization rate; proportion of low-, medium-, or high-quality embryos generated; mean number of oocytes retrieved; or mean number of zygotes generated with two pronuclei. However, despite the (+)GH and (+)GH–DHEA groups being significantly older and having a significantly lower AMH in comparison to the (−)Adj group, they achieved greater oocyte and embryo utilization rates*.

*AMH, anti-Mullerian hormone; BMI, body mass index; DHEA, dehydroepiandrosterone; ET, embryo transfer; GH, growth hormone*.

From the included patient cohort, there was no significant difference between the groups with regard to the mean BMI; fertilization rate; number of embryos cryopreserved; and proportion of high-, medium-, or low-quality embryos generated after OPU (Table 2). However, there were significant differences between the adjuvant treatment groups and (−)Adj for age [younger in (−)Adj], AMH [higher in (−)Adj], mean embryos transferred [fewer in (−)Adj], mean oocytes retrieved [more in (−)Adj], mean two pronuclei generated [more in (−)Adj], and utilization rates [lower in (−)Adj; Table 2].

### Univariate and Multivariate Analysis Using Logistic Regression

Logistic regression models were generated to determine the influence of each individual variable on clinical pregnancy and live birth ORs. Only patient age, transferred embryo development stage (blastocyst versus cleavage stage), transferred embryo quality, and the adjuvant treatment type were significant predictors of clinical pregnancy and/or live birth chance. AFC only affected pregnancy rates significantly in the lower AFC category (≤4 follicles). Patient AMH, BMI, number of embryos transferred, stimulation protocol type, infertility factors, or previous IVF attempts did not influence clinical pregnancy and/or live birth chance significantly or independently (Table 3). When stepwise multiple logistic regression was performed using all terms, only patient age, transferred embryo quality, and adjuvant treatment type were retained and were each independently significant; thus, they were the most important parameters for primary outcome prediction. AFC was not significant for clinical pregnancy or live birth chance in the multivariable model. Overall, increasing patient age decreased the chance of clinical pregnancy and/or live birth by about 10% per advancing year. Following adjustment for age, AFC, and adjuvant treatment type, transferred embryo quality was an independent predictor of clinical pregnancy and live birth, with greatest ORs observed when high-quality day 3 or high-/medium-quality blastocysts were transferred (Table 3). Adjuvant treatment type was also an independent predictor of clinical pregnancy and live birth chance following adjustment for patient age, AFC, and transferred embryo quality, with the largest ORs observed for (+)GH [OR, 3.28 (*p* < 0.000) and 7.07 (*p* < 0.000) for pregnancy and live birth, respectively]. Conversely, (+)DHEA alone did not alter the chance of clinical pregnancy or live birth, with (−)Adj as a reference comparator. Addition of DHEA to GH cycles did not alter the significance of (+)GH–DHEA ORs for clinical pregnancy or live births, with the positive effect from GH still observed [OR, 2.89 (*p* = 0.001) and 5.64 (*p* < 0.000) for pregnancy and live birth, respectively].

**Table 3:** Logistic regression analysis of cycles.

Variable	Clinical pregnancy chance				Live birth chance			
	Univariate (unadjusted)	*p* value	Multivariable	*p* value	Univariate (unadjusted)	*p* value	Multivariable	*p* value
**Adjuvant type**								
(−)Adj	1.00	–	1.00	–	1.00	–	1.00	–
(+)GH	2.49 (1.42–4.37)	0.002	3.28 (1.78–6.01)	0.000	4.75 (2.30–9.79)	0.000	7.07 (3.24–15.41)	0.000
(+)DHEA	0.69 (0.20–2.40)	0.559	0.96 (0.27–3.47)	0.950	NC	NC	NC	NC
(+)GH–DHEA	1.75 (0.98–3.10)	0.058	2.89 (1.54–5.42)	0.001	3.11 (1.48–6.53)	0.003	5.64 (2.52–12.64)	0.000
Age	0.90 (0.86–0.95)	0.000	0.90 (0.85–0.95)	0.000	0.89 (0.84–0.94)	0.000	0.86 (0.81–0.92)	0.000
Serum AMH	1.02 (0.99–1.05)	0.291	–	–	1.02 (0.98–1.06)	0.286	–	–
BMI	1.05 (1.00–1.10)	0.056	–	–	1.01 (0.96–1.07)	0.716	–	–
Number of embryos transferred	1.26 (0.81–1.94)	0.303	–	–	1.25 (0.76–2.06)	0.386	–	–

**AFC grouping (follicle)**								
A (≥20)	1.00	–	1.00	–	1.00	–	1.00	–
B (13–19)	0.67 (0.29–1.53)	0.338	0.86 (0.35–2.12)	0.749	0.93 (0.36–2.39)	0.883	1.20 (0.42–3.40)	0.739
C (9–12)	0.93 (0.44–1.96)	0.841	1.44 (0.61–3.37)	0.405	0.92 (0.38–2.26)	0.857	1.15 (0.40–3.28)	0.791
D (5–8)	0.73 (0.37–1.45)	0.368	1.32 (0.59–2.95)	0.502	0.84 (0.37–1.90)	0.672	1.43 (0.55–3.72)	0.466
E (≤4)	0.28 (0.11–0.72)	0.008	0.57 (0.20–1.61)	0.290	0.35 (0.12–1.04)	0.059	0.71 (0.21–2.42)	0.587

**Stimulation protocol**								
Antagonist cycle	1.00	–	–	–	1.00	–	–	–
Agonist cycle	0.98 (0.60–1.60)	0.936	–	–	0.95 (0.54–1.66)	0.859	–	–
Other cycle (downregulation)	0.95 (0.52–1.75)	0.873	–	–	0.64 (0.30–1.40)	0.266	–	–
Blastocyst versus cleavage								
Cleavage	1.00	–	–	–	1.00	–	–	–
Blastocyst	3.25 (1.81–5.85)	0.000	–	–	2.93 (1.51–5.68)	0.001	–	–

**Quality of transferred embryo**								
Low-quality D3	1.00	–	1.00	–	1.00	–	1.00	–
High-quality blastocyst	5.55 (2.33–13.21)	0.000	5.67 (2.06–15.60)	0.001	3.75 (1.26–10.98)	0.017	3.19 (0.89–11.50)	0.076
Medium-quality blastocyst	3.54 (1.29–9.72)	0.014	3.55 (1.21–10.46)	0.021	4.82 (1.60 14.55)	0.005	5.26 (1.54–17.97)	0.008
Low-quality blastocyst	3.14 (0.81–12.25)	0.099	2.08 (0.47–9.17)	0.334	5.46 (1.36–21.95)	0.017	3.36 (0.69–16.38)	0.134
High-quality D3	1.90 (1.15–3.13)	0.012	1.80 (1.06–3.06)	0.030	2.48 (1.36–4.52)	0.003	2.45 (1.28–4.70)	0.007

**Number of infertility factors**								
None or one factor	1.00	–	–	–	1.00	–	–	–
Two factors	0.66 (0.41–1.04)	0.075	–	–	0.72 (0.42–1.23)	0.228	–	–
Three or more factors	0.61 (0.27–1.38)	0.235	–	–	0.70 (0.28–1.76)	0.445	–	–

**Number of previous IVF attempts**								
No previous attempts	1.00	–	–	–	1.00	–	–	–
One previous attempts	1.37 (0.82–2.32)	0.233	–	–	1.00 (0.54–1.83)	0.994	–	–
Two previous attempts	1.51 (0.73–3.11)	0.268	–	–	0.86 (0.34–2.18)	0.750	–	–
Three or more previous attempts	1.25 (0.63–2.46)	0.525	–	–	1.32 (0.64–2.73)	0.460	–	–

*AFC, antral follicle count; AMH, anti-Mullerian hormone; BMI, body mass index; DHEA, dehydroepiandrosterone; GH, growth hormone; IVF, in vitro fertilization; NC, not computed due to low case number*.

*The presence of GH, patient age, transferred embryo development stage, and quality were the only significant variables that affected clinical pregnancy or live birth chance. When adjusting for these variables in a multivariate logistic analysis, the effect of each parameter became stronger, as reflected by increased odds ratios*.

### Analysis According to Patient Age and Adjuvant Treatment

Since the majority of patients (78.6%) in this poor prognosis cohort were aged between 35 and 44 years, our analysis focused on two specific age groups, 35–39 years and 40–44 years (Table 4). In women aged 35–39 years, (+)GH led to significantly more clinical pregnancies and live births (OR, 4.50; 95% CI, 1.81–11.15 and 14.68, 95% CI, 3.14–68.76, *p* = 0.001, respectively). There was a trend for increased clinical pregnancies in the (+)DHEA group (OR, 2.12; 95% CI, 0.40–11.38; *p* = 0.379), but it was not significant. The case number leading to live births in this group was too few to analyze. However, the addition of DHEA in combination with GH did not alter the positive enhancement of clinical pregnancies and live births from GH (OR, 3.50; 95% CI, 1.41–8.65 and OR, 15.50; 95% CI, 3.37–71.28, *p* < 0.007, respectively). On the other hand, (+)GH was the only adjuvant group to demonstrate any advantage in women aged 40–44 years, showing that there was a significant increase in live birth chance (OR, 5.79; 95% CI, 1.23–27.80; *p* = 0.027; Table 4). This was reflected in the specific age range of 40–41 years (Table 4).

**Table 4:** Logistic regression analysis of age interaction with GH.

	No clinical pregnancy, N (%)	Yes clinical pregnancy, N (%)	Clinical pregnancy, odds ratio (95% CI)	*p* value	Yes live birth, N(%)	Live birth, odds ratio (95% CI)	*p* value
**Unadjusted analysis**							

(−)GH, N (%)	215 (90.0)	24 (10.0)	1.00	–	11 (4.6)	1.00	–
(+)GH, N (%)	126 (78.3)	35 (21.7)	2.49 (1.42–4.37)	0.002	30 (18.6)	4.75 (2.30–9.79)	0.000
(+)DHEA, N (%)	39 (92.8)	3 (7.1)	0.69 (0.20–2.40)	0.559	0 (0.0)	NC	NC
(+)GH–DHEA, N (%)	154 (83.7)	30 (16.3)	1.75 (0.98–3.10)	0.058	24 (13.0)	3.11 (1.48–6.53)	0.003

**Analysis according to age group**							

**Age < 35 years**							
(−)GH, N (%)	47 (82.5)	10 (17.5)	1.00	–	7 (12.3)	1.00	–
(+)GH, N (%)	15 (65.2)	8 (34.8)	2.51 (0.84–7.50)	0.100	8 (34.8)	3.81 (1.19–12.24)	0.025
(+)DHEA, N (%)	6 (100.0)	0 (0.0)	NC	NC	0 (0.0)	NC	NC
(+)GH–DHEA, N (%)	17 (77.3)	5 (22.7)	1.38 (0.41–4.63)	0.599	5 (22.7)	2.10 (0.59–7.50)	0.253

**Age 35–39 years**							
(−)GH, N (%)	86 (90.5)	9 (9.5)	1.00	–	2 (2.1)	1.00	–
(+)GH, N (%)	34 (68.0)	16 (32.0)	4.50 (1.81–11.15)	0.001	12 (24.0)	14.68 (3.14–68.76)	0.001
(+)DHEA, N (%)	9 (81.8)	2 (18.2)	2.12 (0.40–11.38)	0.379	0 (0.0)	NC	NC
(+)GH–DHEA, N (%)	41 (73.2)	15 (26.8)	3.50 (1.41–8.65)	0.007	14 (25.0)	15.50 (3.37–71.28)	0.000

**Age 40–44 years**							
(−)GH, N (%)	78 (94.0)	5 (6.0)	1.00	–	2 (2.4)	1.00	–
(+)GH, N (%)	69 (86.3)	11 (13.8)	2.49 (0.82–7.51)	0.106	10 (12.5)	5.79 (1.23–27.30)	0.027
(+)DHEA, N (%)	22 (95.7)	1 (4.3)	0.71 (0.08–6.39)	0.759	0 (0.0)	NC	NC
(+)GH–DHEA, N (%)	84 (89.4)	10 (10.6)	1.86 (0.61–5.67)	0.277	5 (5.3)	2.28 (0.43–12.05)	0.334

**Age 40–41 years**							
(−)GH, N (%)	46 (92.0)	4 (8.0)	1.00	–	2 (4.0)	1.00	–
(+)GH, N (%)	31 (81.6)	7 (18.4)	2.60 (0.70–9.63)	0.153	7 (18.4)	5.42 (1.06–27.80)	0.043
(+)DHEA, N (%)	10 (90.9)	1 (9.1)	1.15 (0.12–11.42)	0.905	0 (0.0)	NC	NC
(+)GH–DHEA, N (%)	29 (87.9)	4 (12.1)	1.59 (0.37–6.84)	0.536	3 (9.1)	2.40 (0.38–15.21)	0.353

*CI, confidence interval; DHEA, dehydroepiandrosterone; GH, growth hormone; NC, not computed due to low case number*.

*No pregnancies recorded over 44 years*.

*The positive effect of GH on clinical pregnancy or live birth chance was clearly dependent on patient age. Those younger than 39 years were more likely to achieve a live birth (+)GH than (−)GH. However, more beneficial GH effects were observed in the 35–39 years group as reflected by increased clinical pregnancy and live birth chance either in the absence or in the presence of DHEA. Limited GH benefits were observed for live birth in those aged 40–44 years and specifically were found in those aged 40 or 41 years*.

### Analysis According to Transferred Embryo Quality and Adjuvant Treatment

One of the most important independent predictors of pregnancy and live birth outcomes was the quality of the transferred embryo as assessed according to morphological grading (53). The majority of women/cycles (90.1%) included the transfer of a day 3 cleavage stage embryo, whereas only 62 women/cycles (9.9%) involved the transfer of a day 5 blastocyst, which is characteristic of a poor prognosis cohort (Table 5). Consequently, we focused on the interaction between transferred day 3 cleavage stage embryo quality and adjuvant treatment type (Table 5). High-quality day 3 embryos with 8+ cells, no fragmentation and early compaction evident, led to greater pregnancy and live birth chance in comparison to low-quality day 3 embryos with slow cleavage and/or >20% fragmentation, without evident compaction (Table 5). The (−)Adj group demonstrated greater pregnancy rates when high-quality day 3 embryos were transferred. However, (+)GH with or without DHEA [i.e., (+)GH–DHEA] produced significantly more live births in comparison to (−)Adj and (+)DHEA when high-quality embryos were transferred (Table 5). Conversely, only (+)GH altered pregnancy and live birth rates when low-quality day 3 embryos were transferred (OR, 4.69; 95% CI, 1.43–15.37, *p* = 0.011, and OR, 4.38; 95% CI, 1.12–17.09, *p* = 0.034, respectively). No other treatment group demonstrated increased clinical pregnancy rates with low-quality day 3 embryos (Table 5).

**Table 5:** Logistic regression analysis of transferred embryo quality interaction with GH.

	No clinical pregnancy, n (%)	Yes clinical pregnancy, n (%)	Clinical pregnancy, odds ratio (95% CI)	*p* value	Yes live birth, n (%)	Live birth, odds ratio (95% CI)	*p* value
**Unadjusted analysis**							

(-)GH, N (%)	215 (90.0)	24 (10.0)	1.00	-	11 (4.6)	1.00	-
(+)GH, N (%)	126 (78.3)	35 (21.7)	2.49 (1.42–4.37)	0.002	30 (18.6)	4.75 (2.30–9.79)	0.000
(+)DHEA, N (%)	39 (92.8)	3 (7.1)	0.69 (0.20–2.40)	0.559	0 (0.0)	NC	NC
(+)GH-DHEA, N (%)	154 (83.7)	30 (16.3)	1.75 (0.98–3.10)	0.058	24 (13.0)	3.11 (1.48–6.53)	0.003

**Analysis According to transferred embryo quality**							

**High-quality day 3 embryo**							
(−)GH, N (%)	87 (86.1)	14 (13.9)	1.00	–	7 (6.9)	1.00	–
(+)GH, N (%)	57 (77.0)	17 (23.0)	1.85 (0.85–4.05)	0.122	16 (21.6)	3.70 (1.44–9.55)	0.007
(+)DHEA, N (%)	18 (100.0)	0 (0.0)	NC	NC	0 (0.0)	NC	NC
(+)GH–DHEA, N (%)	47 (81.0)	11 (19.0)	1.45 (0.61–3.46)	0.396	10 (17.2)	2.80 (1.00–7.81)	0.050

**Low-quality day 3 embryo**							
(−)GH, N (%)	104 (96.3)	4 (3.7)	1.00	–	3 (2.8)	1.00	–
(+)GH, N (%)	61 (84.7)	11 (15.3)	4.69 (1.43–15.37)	0.011	8 (11.1)	4.38 (1.12–17.09)	0.034
(+)DHEA, N (%)	18 (85.7)	3 (14.3)	4.33 (0.89–21.00)	0.069	0 (0.0)	NC	NC
(+)GH–DHEA, N (%)	100 (89.3)	12 (10.7)	3.12 (0.97–10.00)	0.055	7 (6.3)	2.33 (0.59–9.27)	0.229

*CI, confidence interval; NC, not computed due to low case number*.

*The positive effect of GH on clinical pregnancy or live birth chance was clearly dependent on the quality of transferred embryos. (+)GH increased the clinical pregnancy as well as live birth chance when lower quality Day-3 embryos were transferred, but only improved live birth chance when high quality Day-3 embryos were transferred. However, GH in combination with DHEA had little impact on these outcomes*.

## Discussion

This retrospective study echoed our previous study (10) and showed that patient age, the quality of transferred embryos, and the utilization of GH (with or without DHEA) were significant predictors of clinical pregnancy and live birth rates in IVF patients categorized as poor prognosis. Importantly, following on from our previous study which focused solely on GH (10), the current investigation demonstrated that DHEA administration alone did not significantly influence primary outcomes in comparison to (−)Adj, and it did not alter nor enhance the positive effects from GH therapy when administered in combination. Generally, addition of DHEA to GH cycles led to a decrease in ORs, but the rates and ORs were still more beneficial in comparison to (−)Adj or (+)DHEA therapy alone. Furthermore, DHEA did not affect other secondary parameters such as number of oocytes retrieved or fertilization rates as previously reported (27, 32, 35, 36). Contrary to other reports (25–34), these findings question the use of DHEA as an adjuvant in IVF, but reiterate the potential beneficial effects of GH.

While we acknowledge that the (+)DHEA alone group contained the smallest number of cases, the evidence suggested that DHEA did not convey any beneficial effect as an IVF adjuvant, including with GH, and these data build upon the only other report to investigate the impact of DHEA and GH combinations in IVF (45). In their study of 85 women receiving 183 IVF cycles, Haydardedeoğlu et al. demonstrated that DHEA for 12 weeks, with at least 4 weeks of transdermal testosterone and a late-luteal administration of GH in the previous cycle prior to stimulation, resulted in greater clinical pregnancy and live birth rates, along with more follicles, oocytes retrieved, and higher fertilization rate. There was no difference reported in the morphological quality of developing embryos (45). In our analysis, we also observed differences in pregnancy and live birth rates (45). We also found no significant change in the number of oocytes retrieved nor the fertilization rate. The number of zygotes with two pronuclei developing was lower with (+)GH–DHEA, but not significant, although it may reflect the significantly increased age of the women undertaking these combination adjuvant cycles in our study. Similar to the aforementioned study, we also observed no significant difference in embryo quality as assessed by the morphological analysis. However, they used a complicated treatment strategy that involved three different adjuvant agents, and the treatment group also consisted of only 37 cycles (45). Therefore, our combination study may indeed be considered superior to the study by Haydardedeoğlu et al., until the data become available for the combined GH–DHEA trial NCT02766764, which is currently recruiting.

Importantly, the (+)GH results in this study also reflect our earlier work (13) and comparable data derived from an RCT by Tesarik et al. (16), which suggested that GH lowered miscarriage rate leading to more live births. There are some notable differences between the studies such as patients in the study by Tesarik et al. being older with a mean age of 42 years and the researchers transferring three to four embryos. In terms of the ORs for clinical pregnancy and live births in this study, similar ORs were observed in several meta-analyses (12, 15, 17). However, other recent meta-analyses indicated that GH had no significant influence on live birth, with lower ORs (7, 8), but a slight increase in pregnancy rate (8).

The main aim of this study is to retrospectively examine in one of the largest adjuvant cohorts, the impact of adjuvant therapy on clinical IVF outcomes. Another crucial aim is to identify critical confounding variables and participant inclusion characteristics outside of the Bologna criteria that could be considered when designing future IVF adjuvant RCTs. The most important confounding variables we identified were patient age and quality of the transferred embryo, which is supported by our previous report (10). In this analysis, we showed that additional patient characteristics such as AMH, BMI, number of infertility factors, previous IVF attempts, or mean number of embryos transferred did not have an independent effect on clinical pregnancy or live birth chance and may be related to our specific rFSH dosing algorithms (48, 54).

The patients in our analysis were categorized as poor prognosis, with low AFC (≤8 follicles) and significantly reduced serum AMH level. Those treated with (+)GH, (+)DHEA, and (+)GH–DHEA were older on average with a lower AMH in comparison to those (−)Adj. In spite of this perceived very poor ovarian reserve and advanced maternal age, we showed that when (+)GH was added to cycles, with or without DHEA, the oocyte and embryo utilization rates, along with pregnancy and live births rates, were greater. We also investigated the effect of (+)GH on patients with different AFC gradings, but neither AFC nor the presence or absence of GH or DHEA significantly altered clinical pregnancy or live birth chance in different AFC groupings. Notably, because patient ovarian reserve has not been described in any other IVF study utilizing GH (17), direct comparison of our findings is restricted. However, AFC as a specific marker of ovarian reserve was insignificant following adjustment for patient age. In relation to age, we did not observe any benefit in patients older than 41 years. Taken together, these findings have implications for these specific parameters within the Bologna criteria. Therefore, newer adjuvant studies should probably focus on women 40 years or below, who generate lower quality embryos with standard stimulation and with RIF, rather than those defined by the Bologna criteria.

There are significant strengths associated with this study including the use of a large data set (n = 626 cycles), a low number of transferred embryos per cycle (average 1.5/cycle), and incorporation of several potential confounding variables such as age, AFC, AMH, and transferred embryo quality. However, like all retrospective analyses and most GH and DHEA studies in the IVF research space, it has significant limitations that should be cautiously considered when interpreting the findings. The potential positive effects of GH and the disappointing effects of DHEA are strictly associative rather than causative, as this study was not designed as an intervention RCT. In addition, given the retrospective design, the study is also subject to significant patient selection bias and the process by which the control group [i.e., (−)Adj] was selected must be carefully understood. Justifiably, it was decided to generate this group using treatment cycles free from adjuvant intervention, but to only include these adjuvant-free cycles from women who received adjuvant therapy during their entire treatment history. This meant that the group comprised only women considered poor prognosis, being offered adjuvant therapy due to one or more of the five reasons outlined in Section “Materials and Methods.” In an attempt to randomize cases and to counter any other perceived selection bias, we chose to analyze only the very first IVF cycle with fresh embryo transfer for each women during the study period, regardless of whether there was an adjuvant intervention. This process also prevented any confounding that may be interpreted from including multiple treatment cycles for individual women.

In addition, there is a significant heterogeneity in terms of poor prognosis factors and combination thereof in the adjuvant treatment groups. While the adjuvant groups, especially GH, tended to have a higher proportion of patients with these factors, the heterogeneity is an important limitation of this study. Future studies must focus on more homogenous groups, but as indicated here, these could incorporate factors outside of the traditional Bologna criteria and possibly have a more nuanced approach like that of the POSEIDON study (55). Other factors that may be important but not considered in the current analysis was patient socioeconomic status and parity. Socioeconomic status and affordability could be critical confounders as patients were required to pay for IVF adjuvants. Taken together, while not as robustly designed and as powerful as a prospective RCT, the current study design limited any perceived bias and is one of the largest GH studies in IVF research to date.

Overall, we have shown for the first time in a large data set, and adjusting for various confounding variables, that (+)GH alone or in combination with DHEA, significantly increased the chance of clinical pregnancy and live birth outcomes in poor prognosis IVF patients, who tended to be significantly older (1.5–2.0 years) and had a lower average serum AMH value. This new retrospective adjuvant IVF study with GH and DHEA alone and in combination is the first to include aspects of analysis such as AFC, AMH, BMI, and embryo quality assessment and has provided further evidence to indicate the potential beneficial effects of GH supplementation in IVF treatment. It also demonstrated that DHEA did not appear to have a significant influence on primary IVF outcomes. Although the study has certain limitations in that it is observational and retrospective in nature, bias was minimized as best as possible, and the data suggested that GH supplementation may provide more live births, mainly in younger women and questions the use of adjuvant therapy in women older than 41 years. Finally, the data indicate that future GH and DHEA IVF RCTs should strictly account for transferred embryo quality and expectedly age, while possibly focusing on younger women with RIF or those generating a greater proportion of low-quality embryos.

## Ethics Statement

Our clinic is accredited with the Reproductive Technology Accreditation Committee, a National body, as well as the Reproductive Technology Council of Western Australia, a State body. These agencies monitor all activities according to respective Codes of Practice. Specific ethics approval was not required for this study as all procedures and blood tests were embraced by routine approved clinical protocols. However, retrospective analysis and reporting of the data was approved under Curtin University Ethics Committee approval no. RD_25-10. In addition, as a part of our documentation system, written informed-consent was obtained from each participant who accepted the use of adjuvants, and they were required to pay for these adjuvants over and above the IVF treatment charges.

## Author Contributions

The present work was designed by JY, KK, and SD. Data extraction and analysis were performed by KK, PH, SD, and JY. Patient recruitment was undertaken by PR, GB, and SS. The initial manuscript draft was prepared by KK and subsequently revised by JY, SD, and PH. All the authors approved the final submitted version.

## Conflict of Interest Statement

All authors have nothing to disclose and approve the submitted copy of the manuscript. KK is employed as a Research Fellow at Curtin University and a portion of this position is financially supported by PIVET Medical Centre. PH, PR, GB, and SS provide consultancy services to PIVET Medical Centre. SD is a Curtin University Professor and an adjunct Research Consultant at PIVET Medical Centre. JY is the salaried Medical Director of PIVET Medical Centre and an adjunct Clinical Professor at Curtin University.
